# Forward Brillouin scattering in few-mode fibers

**DOI:** 10.1038/s41377-025-01877-z

**Published:** 2025-07-09

**Authors:** Elad Layosh, Elad Zehavi, Alon Bernstein, Mirit Hen, Maayan Holsblat, Ori Pearl, Avi Zadok

**Affiliations:** 1 Faculty of Engineering and Institute for Nano-Technology and Advanced Materials, Bar-Ilan University, Ramat-Gan, 5290002 Israel; 2 Presently with the Faculty of Electrical and Computer Engineering and the Solid State Institute, Technion – Israel Institute of Technology, Haifa, 3200003 Israel

**Keywords:** Nonlinear optics, Fibre optics and optical communications

## Abstract

Forward Brillouin scattering is an opto-mechanical effect in which two co-propagating optical fields couple with an acoustic mode in a common medium. The effect has been studied in standard optical fibers since 1985, however nearly all studies have been limited to the single optical mode regime. Forward Brillouin scattering in single-mode fibers takes place through two classes of acoustic modes only: purely radial ones and modes of two-fold azimuthal symmetry. Acoustic modes may only be stimulated at their cut-off frequencies, and the acoustic frequencies in standard fibers are limited to 600 MHz. In this work, we extend the study of forward Brillouin scattering to few-mode optical fibers through analysis, calculations, and experiment. Measurements are performed in a commercial, off-the-shelf step-index fiber with standard cladding through three optical modes. We demonstrate for the first time the stimulation of acoustic modes with first-order and fourth-order azimuthal symmetries. Acoustic frequencies up to 1.8 GHz are observed, and the acoustic modes are stimulated above their cut-off frequencies. Angular momentum is transferred between the orbital degrees of freedom of the optical and acoustic waves. The results extend the understanding and formulation of forward Brillouin scattering in optical fibers, and they may find applications in fiber lasers, sensing, non-reciprocal propagation effects, and quantum states manipulation.

## Introduction

Brillouin scattering is an opto-mechanical interaction that couples between traveling optical and acoustic waves in a common medium^1–8^. The phenomenon has been studied in fibers for over 50 years^1–8^. Brillouin scattering in standard fibers may take place in either the backward or forward directions^1–8^. In backward interactions, two counter-propagating optical field components may stimulate a longitudinal acoustic mode that is guided in the core of the fiber^6–8^. In forward Brillouin scattering, two co-propagating optical fields couple with a predominantly transverse acoustic mode that is guided by the entire cladding cross-section^1–5^. Forward Brillouin scattering in single-mode fibers has been formulated and reported in 1985^1^. Interest in the effect has increased in recent years, towards sensing of substances outside the boundaries of standard cladding, where guided light does not reach^5,9–24^. The decay rates of the acoustic modes in forward Brillouin scattering interactions are affected by the elastic boundary conditions at the edge of the cladding, and their monitoring enables the analysis of surrounding media^5,9–24^.

Most studies of forward Brillouin scattering in standard fibers were carried out in the single-mode regime^1–5^. A pair of optical fields in the fundamental, single mode may only stimulate acoustic modes of two specific classes: purely radial ones, and modes of two-fold azimuthal symmetry^1–5^. Although the fiber cladding supports guided acoustic modes of any integer order of azimuthal symmetry, other classes of acoustic modes cannot be addressed through forward Brillouin scattering in single-mode fibers. The efficiency of forward Brillouin scattering scales with the spatial overlap between the transverse profiles of the optical and acoustic modes involved^1–5^. In standard single-mode fibers, that overlap is maximal for acoustic modes of frequencies between 200-600 MHz^1–5^. The effect diminishes strongly at higher acoustic frequencies. Lastly, forward Brillouin stimulation of acoustic modes in single-mode fibers is only possible at their cut-off frequencies^1–5^. At that limit, the acoustic modes are almost entirely transverse, and their axial wavenumbers are vanishingly small^1–5^.

Over the last decade, few-mode optical fibers have taken an increasing role in space-division multiplexing of optical telecommunication channels^25,26^, and they have also found many sensing applications^27^. The number of mode groups supported by the fiber can be controlled through the dimensions and index contrast of the core^28,29^. Few-mode fibers provide additional degrees of freedom for Brillouin scattering interactions, as the optical fields that take part in the process may be guided in different spatial modes. Brillouin scattering interactions through multiple guided optical modes have been reported in photonic integrated waveguides^30–32^ and in thin tapered fibers^33,34^. Backward Brillouin scattering interactions have been investigated in standard few-mode fibers^35,36^, and they were used towards strain and temperature sensing and for modal dispersion analysis^37^. The forward effect has been studied in standard, panda-type polarization-maintaining fibers, in which the two polarization modes are non-degenerate^38^. However, the difference in effective indices between the two principal states of the fiber is comparatively small, in the fourth decimal point. In addition, the guided acoustic modes of the panda-type fiber cannot be solved analytically and do not maintain regular azimuthal symmetries^38^. Forward Brillouin scattering in standard few-mode fibers has yet to be examined.

In this work, we report the analysis, calculations, and experimental demonstration of forward Brillouin scattering in a step-index few-mode fiber with standard cladding. The spectrum of forward Brillouin scattering is formulated for the launch of each of the optical fields involved in an arbitrary guided mode. The experimental setup supported selective coupling of light to the fundamental ${{LP}}_{01}$ mode, the ${{LP}}_{02}$ mode, or the ${{LP}}_{11}$ mode group of a few-mode fiber. The results show that forward Brillouin scattering in the ${{LP}}_{02}$ mode reaches acoustic frequencies up to 1.8 GHz, much higher than in the fundamental mode. The measurements demonstrate the Brillouin stimulation of additional classes of acoustic modes, with first-order and fourth-order azimuthal symmetries, which are inaccessible through the corresponding process in single-mode fibers. Further, inter-modal interactions between optical waves in the ${{LP}}_{01}$ and ${{LP}}_{02}$ modes stimulate acoustic modes that are detuned from their cut-off frequencies by a few MHz. Certain inter-modal forward Brillouin interactions signify the transfer of angular momentum quanta between the orbital degrees of freedom of optical and acoustic waves^39,40^. The results extend the formulation and scope of fiber opto-mechanics beyond the single-mode regime, and they may find applications in fiber lasers, sensing, and quantum states manipulation.

## Results

### Analysis of forward Brillouin scattering in standard few-mode fibers

Solutions to Maxwell’s equations in standard step-index optical fibers are given by a discrete set of optical modes, denoted by $H{E}_{{\it ln}}$ and $E{H}_{{\it ln}}$ [29]. The first integer index $l\ge 0$ denotes the order of azimuthal symmetry of a given solution, whereas the second integer $n\ge 1$ refers to the radial order. In the $H{E}_{{\it ln}}$ modes the axial component of the magnetic field is larger than that of the electric field. The opposite is true for the $E{H}_{{ln}}$ modes. In both categories, the axial field components are much smaller than the transverse ones. For $l=0$ the solutions are referred to as $T{E}_{0n}$ and $T{M}_{0n}$ modes, in which the transverse vector profile of the electric field is purely radial (for $T{M}_{0n}$) or purely torsional (for $T{E}_{0n}$).

The exact modal solutions may be classified in groups which share the same transverse radial profiles. These mode groups are referred to as the linearly polarized (LP) modes $L{P}_{{\it ln}}$ [29]. Each $L{P}_{0n}$ group consists of the $H{E}_{1n}$ mode only, and each $L{P}_{1n}$ group includes the $T{E}_{0n}$, $T{M}_{0n}$ and $H{E}_{2n}$ modes. For $l\ge 2$, each $L{P}_{{\it ln}}$ group consists of the $H{E}_{l+1,n}$ and $E{H}_{l-1,n}$ modes. The differences in the refractive indices among modes within the same LP group are in the order of 10⁻⁵ refractive index units (RIU), 2-3 orders of magnitude smaller than the corresponding differences between different LP mode groups. In the following we disregard the small differences between effective indices of modes in the same LP group.

Let us denote the axial propagation constant of optical modes group $L{P}_{{\it ln}}$ as ${\beta }_{{\it ln}}$, and define: ${h}_{{\it ln}}^{{core}}=\sqrt{{n}_{1}^{2}{k}_{0}^{2}-{\beta }_{{\it ln}}^{2}}$ and ${h}_{{\it ln}}^{{clad}}=\sqrt{{\beta }_{{\it ln}}^{2}-{n}_{2}^{2}{k}_{0}^{2}}$. Here ${n}_{\mathrm{1,2}}$ are the refractive indices of the uniform core and cladding, respectively, and ${k}_{0}$ is the vacuum wavenumber of incident light. Within the weak guiding approximation, which is valid for standard fibers, the radial profile of the optical field in group $L{P}_{{\it ln}}$ is given by

1 $${G}_{{ln}}\left(r\right)={E}_{{ln}}\left\{\begin{array}{c}\frac{{J}_{l}\left({h}_{{ln}}^{{core}}r\right)}{{h}_{{ln}}^{{core}}{J}_{l+1}({h}_{{ln}}^{{core}}a)}{{r}}\le {a}\\ \frac{{K}_{l}\left({h}_{{ln}}^{{clad}}r\right)}{{h}_{{ln}}^{{clad}}{K}_{l+1}({h}_{{ln}}^{{clad}}a)}{r} \,>\, {a}\end{array}\right.$$

In Eq. (1), $r$ is the radial transverse coordinate, $a$ denotes the core radius, ${J}_{l}$ is the Bessel function of the first kind, order $l$, and ${K}_{l}$ represents the modified Bessel function of the second kind, order $l$. The factor ${E}_{{\it ln}}$ is a normalization constant (see below). The normalized transverse profiles of the electric field vectors in the guided optical core modes of the fiber are expressed as

2 $${\vec{E}}_{T,\mathrm{\it ln}}^{({HE})}(r,\phi )={G}_{l-1,n}\left(r\right)\left[\left\{\begin{array}{c}\cos \left(l\phi\right)\\ \sin \left(l\phi \right)\end{array}\right\}\hat{{\boldsymbol{r}}}+\left\{\begin{array}{c}-\sin \left(l\phi \right)\\ \cos \left(l\phi \right)\end{array}\right\}\hat{{\boldsymbol{\phi }}}\right]$$

3 $$\begin{array}{l}{\vec{E}}_{T,\mathrm{\it ln}}^{({EH})}(r,\phi )={G}_{l+1,n}\left(r\right)\left[\left\{\begin{array}{c}\cos \left(l\phi \right)\\ \sin \left(l\phi \right)\end{array}\right\}\hat{{\boldsymbol{r}}}\right.\\\qquad\qquad\quad\left.+\left\{\begin{array}{l}\sin \left(l\phi \right)\\ -\cos \left(l\phi \right)\end{array}\right\}\hat{{\boldsymbol{\phi }}}\right]\end{array}$$

The expressions in Eqs. (2) and (3) relate to the $H{E}_{{\it ln}}$ and $E{H}_{{\it ln}}$ modes, respectively. In the above equations $\phi$ denotes the transverse azimuthal coordinate and $\hat{{\boldsymbol{r}}}$, $\hat{{\boldsymbol{\phi }}}$ are unit vectors in the radial and azimuthal directions, respectively. Each mode includes two spatially orthogonal solutions, denoted in the curled brackets. The normalization constants ${E}_{{\it ln}}$ in Eq. (1) are defined so that $\iint {\left|{\vec{E}}_{T,\mathrm{\it ln}}^{\left({HE}\right),\left({EH}\right)}\left(r,\phi \right)\right|}^{2}{rdrd}\phi =1$. The units of ${G}_{{\it ln}}$ (and of ${\vec{E}}_{T,\mathrm{\it ln}}^{\left({HE}\right),\left({EH}\right)}$) are m^−1^.

Consider two co-propagating optical fields of optical frequencies ${\omega }_{\mathrm{1,2}}={\omega }_{{\rm{p}}}\,\pm\, \tfrac{1}{2}\Omega$, in spatial modes 1 and 2, respectively,

4 $${\vec{E}}_{1,2}\left(r,\phi ,z,t\right)={A}_{1,2}\left(z\right){\vec{E}}_{T,1,2}(r,\phi )\exp \left(j{\beta }_{1,2}z-j{\omega }_{1,2}t\right)+c.c$$

Here ${\omega }_{p}$ is a central optical frequency, $\Omega$ denotes a frequency detuning on the acoustic scale, ${A}_{1,2}\left(z\right)$ represent scalar complex magnitudes in Volts that may vary with axial position $z$, and $t$ stands for time. ${\beta }_{1,2}$ and ${\vec{E}}_{T1,2}$ are the propagation constants and normalized transverse profiles of the two waves, according to their specific modes. The electro-strictive stress tensor ${{\boldsymbol{\sigma }}}_{\mathrm{1,2}}$ [N × m^−2^] associated with the two fields in spatial modes 1 and 2 includes components which propagate along the fiber axis with the frequency difference $\Omega$ and an axial wavenumber $K={\beta }_{1}-{\beta }_{2}$. These components of the stress tensor are given by^41^

5 $$\left(\begin{array}{c}{\sigma }_{{rr},1,2}\left(r,\phi ,z,t\right)\\ {\sigma }_{\phi \phi ,1,2}\left(r,\phi ,z,t\right)\\ {\sigma }_{r\phi ,1,2}\left(r,\phi ,z,t\right)\end{array}\right)=-\frac{1}{4{n}_{0}c}{n}_{0}^{4}\left(\begin{array}{ccc}{p}_{11} & {p}_{12} & 0\\ {p}_{12} & {p}_{11} & 0\\ 0 & 0 & {p}_{44}\end{array}\right)\left(\begin{array}{c}{E}_{r,1}\left(r,\phi \right){E}_{r,2}\left(r,\phi \right)\\ {E}_{\phi ,1}\left(r,\phi \right){E}_{\phi ,2}\left(r,\phi \right)\\ {E}_{r,1}\left(r,\phi \right){E}_{\phi ,2}\left(r,\phi \right)+{E}_{\phi ,1}\left(r,\phi \right){E}_{r,2}\left(r,\phi \right)\end{array}\right)\times P\left(\Omega ,z\right)\exp \left({jKz}-j\Omega t\right)+c.c$$

In Eq. (5), ${p}_{11},{p}_{12}$ and ${p}_{44}=\frac{1}{2}({p}_{11}-{p}_{12})$ are unitless photoelastic parameters of silica, ${n}_{0}\approx {n}_{\mathrm{1,2}}$ is the refractive index of silica, and $c$ denotes the speed of light in vacuum. $P\left(\Omega ,z\right)=2{nc}{\varepsilon }_{0}{A}_{1}\left(z\right){A}_{2}^{* }\left(z\right)$ [W] refers to the beating power between the two optical waves, where ${\varepsilon }_{0}$ is the vacuum permittivity. Lastly, ${E}_{r,\mathrm{1,2}}$ denote the radial components of ${\vec{E}}_{T,1,2}$, and ${E}_{\phi ,\mathrm{1,2}}$ are the corresponding azimuthal components. The electro-strictive driving force per unit volume ${\vec{F}}_{\mathrm{1,2}}$ [N × m^−3^], induced by the pair of fields in modes 1 and 2, is derived from the stress tensor^41^:

6 $$\begin{array}{l}{\vec{F}}_{1,2}\left(r,\phi ,z,t\right)=-\left[\frac{\partial {\sigma }_{{rr},1,2}}{\partial r}+\frac{1}{r}\frac{\partial {\sigma }_{r\phi ,1,2}}{\partial \phi }+\frac{1}{r}\left({\sigma }_{{rr},1,2}-{\sigma }_{\phi \phi ,1,2}\right)\right]\hat{{\boldsymbol{r}}}\\\qquad\qquad\qquad\quad-\,\left[\frac{\partial {\sigma }_{r\phi ,1,2}}{\partial r}+\frac{1}{r}\frac{\partial {\sigma }_{\phi \phi ,1,2}}{\partial \phi }+\frac{2}{r}{\sigma }_{r\phi ,1,2}\right]\hat{{\boldsymbol{\phi }}}\end{array}$$

The expression may be rearranged as

7 $${\vec{F}}_{1,2}\left(r,\phi ,z,t\right)=\frac{1}{4{n}_{0}c}{\vec{f}}_{1,2}\left(r,\phi \right)P\left(\Omega ,z\right)\exp \left({jKz}-j\Omega t\right)+c.c$$

The vector ${\vec{f}}_{\mathrm{1,2}}\left(r,\phi \right)$ (units of m^−3^) contains the transverse dependence of the electro-strictive force per unit volume. Examination of Eq. (7) reveals that the electro-strictive force per unit volume $\vec{F}$ propagates along the fiber axis as a wave, whose magnitude scales with the beating power $P$ between the two optical fields. The frequency of that wave equals the difference $\Omega$ between the two optical frequencies, and its axial wavenumber $K$ is the difference between the two optical propagation constants. Further, substitution of Eqs. (2) and (3) into Eqs. (5) and (6) reveals that the electro-strictive force includes terms with azimuthal orders ${l}_{1}\pm {l}_{2}$ only, where ${l}_{\mathrm{1,2}}$ are the azimuthal orders of the two optical fields. For example, when both optical waves are guided in the fundamental mode $L{P}_{01}$ (${l}_{\mathrm{1,2}}=1$), the electro-strictive force per unit volume consists of a radially symmetric term and a term of two-fold azimuthal symmetry. Expressions for the specific case are given in many references^1–5^. By contrast, if the optical fields 1 and 2 propagate in arbitrary high-order modes, the electro-strictive force may take up any integer order of azimuthal symmetry.

The axial wavenumber of the electro-strictive force per unit volume varies considerably between intra-modal processes, in which the two optical fields propagate in the same spatial mode, and inter-modal processes in which the two modes are different. In the intra-modal case, $K={n}_{{\rm{eff}},1}\Omega /c$, where ${n}_{{\rm{eff}},1}$ is the effective index of the common optical mode. That wavenumber is very small, typically in the order of 1–10 rad × m^−1^. By contrast, in inter-modal interactions we find $K={n}_{{\rm{eff}},1}({\omega }_{{\rm{p}}}+\frac{1}{2}\Omega )/c-{n}_{{\rm{eff}},2}({\omega }_{{\rm{p}}}-\frac{1}{2}\Omega )/c\approx ({n}_{{\rm{eff}},1}-{n}_{{\rm{eff}},2}){\omega }_{{\rm{p}}}/c$, where ${n}_{{\rm{eff}},2}$ is the effective index of optical mode 2 [38]. Since the difference in effective indices between mode groups may reach the second decimal point, the axial wavenumber of the electro-strictive force driven by two distinct mode groups may reach the order of 10^4 ^rad × m^−1^. These values may be within a single order of magnitude of the wavenumbers of bulk acoustic waves of frequency $\Omega$ in silica. The large differences in wavenumbers between intra-modal *vs*. inter-modal scattering manifest in the forward Brillouin spectra of the two settings, as discussed later in this section.

The standard cladding of an optical fiber also serves as an acoustic waveguide, supporting several categories of guided acoustic modes^42,43^. Solutions to the elastic wave equations for the boundary conditions of a bare fiber in air are in the form of torsional-radial (TR) modes $T{R}_{{pm}}$, in which the transverse profiles of material displacement are described by an integer azimuthal order $p\ge 0$ and an integer radial order $m\ge 1$. Displacement in modes with $p=0$ is either purely radial, (such modes are denoted by ${R}_{0m}$), or entirely torsional (in so-called ${T}_{0m}$ modes). Unlike the optical modes discussed above, which are highly confined to the core, the acoustic TR modes relevant to forward Brillouin scattering span the entire cross-section of the fiber cladding. (Note, however, that acoustic modes guided by the core of standard fibers exist as well, and so do optical cladding modes that span the entire fiber cross-section. These categories of acoustic core modes and optical cladding modes are not considered in this work.)

Each TR-guided acoustic mode is characterized by a cut-off frequency ${\Omega }_{{pm}}$, below which it may no longer propagate in the axial direction. Close to their cut-offs, the axial wavenumbers of the acoustic modes vanish and their phase velocities in the axial direction approach infinity. At that limit, both the material displacement vectors and the wave vectors of the acoustic modes are almost entirely transverse^42,43^. The cut-off frequency ${\Omega }_{{pm}}$ is given by the ${m}^{{\rm{th}}}$ eigen-value of the boundary conditions equation for a bare fiber cladding in air^1–5^:

8 $$\left|\begin{array}{cc}{p}^{2}-1-\frac{1}{2}{\Psi }^{2} & 2\left({p}^{2}-1\right)\left[{\Theta }_{p}\left(\Psi \right)-p\right]-{\Psi }^{2}\\ {\Theta }_{{\rm{p}}}\left(\Phi \right)-p-1 & 2{p}^{2}-2\left[{\Theta }_{p}\left(\Psi \right)-p\right]-{\Psi }^{2}\end{array}\right|=0$$

9 $$\Psi =\frac{\Omega b}{{v}_{L}};\Phi =\frac{\Omega b}{{v}_{S}};{\Theta }_{{\rm{p}}}\left(\xi \right)=\frac{\xi {J}_{p-1}\left(\xi \right)}{{J}_{p}\left(\xi \right)}$$

Here ${v}_{L,S}$ are the velocities of dilatational and shear acoustic waves in silica, respectively, and $b$ denotes the cladding radius. The normalized displacement profile of mode $T{R}_{{pm}}$ is given by^1–5^

10 $${\vec{u}}_{{pm}}\left(r,\phi \right)={D}_{{pm}}\left[{A}_{{pm}}\frac{{\Omega }_{{pm}}}{{v}_{L}}{J}_{p}^{{\prime} }\left(\frac{{\Omega }_{{pm}}}{{v}_{L}}r\right)+\frac{p}{r}{C}_{{pm}}{J}_{p}\left(\frac{{\Omega }_{{pm}}}{{v}_{S}}r\right)\right]\left\{\begin{array}{c}\cos (p\phi )\\ \sin (p\phi )\end{array}\right\}\hat{{\boldsymbol{r}}}\,{{+}}\,{D}_{{pm}}\left[\frac{p}{r}{A}_{{pm},}{J}_{p}\left(\frac{{\Omega }_{{pm}}}{{v}_{L}}r\right)+{C}_{{pm}}\frac{{\Omega }_{{pm}}}{{v}_{S}}{J}_{p}^{{\prime} }\left(\frac{{\Omega }_{{pm}}}{{v}_{S}}r\right)\right]\left\{\begin{array}{c}-\sin (p\phi )\\ \cos (p\phi )\end{array}\right\}\hat{{\boldsymbol{\phi }}}$$

The coefficients ${A}_{{pm}}$, ${C}_{{pm}}$ are given by the elements of the eigen-vector corresponding to the eigen-value ${\Omega }_{{pm}}$ of the boundary conditions equation^44^, and the normalization constant ${D}_{{pm}}$ is chosen so that $\iint {\left|{\vec{u}}_{{pm}}\left(r,\phi \right)\right|}^{2}{rdrd}\phi =1$. The units of ${\vec{u}}_{{pm}}$ are therefore m^−1^. The prime superscript represents the derivative of a function with respect to its argument.

The radial and azimuthal components of the normalized displacement in Eq. (10) consist of two terms each. The first term in each pair describes dilatational wave motion, governed by velocity ${v}_{L}$, whereas the second term in each pair represents shear wave motion with velocity ${v}_{S}$ [44]. Every ${{TR}}_{{pm}}$ mode with $p\ge 1$ includes nonzero contributions of both types of waves. The relative magnitudes of the two contributions are proportional to ${A}_{{pm}}$, ${C}_{{pm}}$. TR modes may be broadly classified as either predominantly dilatational, where ${A}_{{pm}}\gg {C}_{{pm}}$, or shear-like, in cases where ${C}_{{pm}}\gg {A}_{{pm}}$ [17,18,44]. Monitoring both classes of TR modes extends sensing applications of forward Brillouin scattering^44^. The radial modes ${R}_{0m}$ are purely dilatational, whereas the ${T}_{0m}$ modes are strictly shear waves. The spacing between the cut-off frequencies ${\Omega }_{{pm}}$ of TR modes of the same order $p$ that are primarily dilatational is approximately ${v}_{L}/\left(2b\right)$, and the corresponding spacing for shear-like modes equals approximately ${v}_{S}/\left(2b\right)$ [17,18]. While the classification of modes as either dilatational or shear-like is not complete, and the above frequency spacings are not precise, they are nevertheless useful in the study of forward Brillouin scattering processes^17,18,44^.

Close to cut-off, the dispersion relations between axial wavenumber $K$ and frequency $\Omega$ of dilatational or shear-dominated modes may be approximated by^1–5^

11 $$\Omega -{\Omega }_{{pm}}\approx \frac{{v}_{L,S}^{2}}{2{\Omega }_{{pm}}}{K}^{2}$$

The detuning from cut-off may reach several MHz for the $K$ values of inter-modal forward Brillouin stimulation.

The electro-strictive force per unit volume may stimulate the propagation of guided acoustic modes of the same frequency $\Omega$ and wavenumber $K$. The displacement in [m] of the stimulated acoustic wave can be expressed as

12 $${\mathop{U}\limits^{ \rightharpoonup }}_{{pm}}\left(r,\phi ,z,t\right)={b}_{{pm}}\left(\Omega ,z\right){\vec{u}}_{{pm}}\left(r,\phi \right)\exp \left({jKz}-j\Omega t\right)+c.c$$

Here ${b}_{{pm}}\left(\Omega ,z\right)$ [m^2^] denotes a modal magnitude which is frequency dependent and may also vary with axial position. The acoustic wave magnitude scales with the beating power between the optical fields $P\left(\Omega ,z\right)$, and with the spatial overlap integral between the transverse profiles of the electro-strictive force per unit volume and the acoustic mode displacement^1–5^:

13 $${Q}_{1,2,{pm}}^{({ES})}=\mathop{\int}\limits_{0}^{{2\pi }}\mathop{\int }\limits_{0}^{{b}}{\vec{u}}_{{pm}}\left(r,\phi \right)\cdot {\vec{f}}_{1,2}\left(r,\phi \right){rdrd}\phi$$

The spatial overlap integral depends on the choices of the optical modes 1 and 2 and of the acoustic mode ${{TR}}_{{pm}}$. The overlap vanishes when the azimuthal orders of ${\vec{u}}_{{pm}}$ and ${\vec{f}}_{\mathrm{1,2}}$ do not match. Consequently, the azimuthal order $p$ of the stimulated acoustic mode must equal ${l}_{1}\pm {l}_{2}$. Referring again to the specific case of two optical fields in the fundamental mode in which ${l}_{\mathrm{1,2}}=1$, we find that only acoustic modes with $p=\mathrm{0,2}$ may be excited through forward Brillouin interactions: ${R}_{0m}$, ${T}_{0m}$, or $T{R}_{2m}$. Further, the azimuthally independent component of ${\vec{f}}_{\mathrm{1,1}}$ in single-mode fiber is entirely in the $\hat{{\boldsymbol{r}}}$ direction^1–5^. Therefore the ${R}_{0m}$ acoustic modes are observed in forward Brillouin scattering in single-mode fibers, but the ${T}_{0m}$ are not.

By contrast, forward Brillouin interactions in few-mode fibers, with one or both optical fields propagating in optical modes with $l\ne 1$, may result in the stimulation of additional classes of guided acoustic modes with different azimuthal symmetries. For example, the ${{LP}}_{\mathrm{1,1}}$ group contains modes with $l=0$ and $l=2$. Electro-strictive stimulation through optical modes within that group may generate acoustic waves with azimuthal orders $p=\mathrm{0,2,4}$. Inter-modal interactions between optical fields in the ${{LP}}_{\mathrm{0,1}}$ mode and the ${{LP}}_{\mathrm{1,1}}$ group might stimulate acoustic modes with $p=1$ or $p=3$.

Material displacement in the stimulated acoustic mode is associated with symmetric strain in the form of a unitless tensor ${{\boldsymbol{S}}}_{{pm}}$:

14 $${{\boldsymbol{S}}}_{{pm}}\left(r,\phi ,z,t\right)={b}_{{pm}}\left(\Omega ,z\right){{\boldsymbol{s}}}_{{pm}}\left(r,\phi \right)\exp \left({jKz}-j\Omega t\right)+c.c$$

The elements of the symmetric normalized strain tensor ${{\boldsymbol{s}}}_{{pm}}\left(r,\phi \right)$, in units of m^−2^, are defined as^42,43^

15 $${s}_{{rr},{pm}}\left(r,\phi \right)=\frac{\partial {u}_{r,{pm}}\left(r,\phi \right)}{\partial r}$$

16 $${s}_{\phi \phi ,{pm}}\left(r,\phi \right)=\frac{{u}_{r,{pm}}\left(r,\phi \right)}{r}+\frac{1}{r}\frac{\partial {u}_{\phi ,{pm}}\left(r,\phi \right)}{\partial \phi }$$

17 $${s}_{r\phi ,{pm}}\left(r,\phi \right)=\frac{1}{2}\left(\frac{1}{r}\frac{\partial {u}_{r,{pm}}\left(r,\phi \right)}{\partial \phi }+\frac{\partial {u}_{\phi ,{pm}}\left(r,\phi \right)}{\partial r}-\frac{{u}_{\phi ,{pm}}\left(r,\phi \right)}{r}\right)$$

In the above relations, ${u}_{r,{pm}}$ and ${u}_{\phi ,{pm}}$ denote the radial and azimuthal components of the normalized displacement vector, respectively (see Eq. (10)). Strain in the fiber medium gives rise to photoelastic perturbations to the dielectric tensor^1–5,41^:

18 $${{\mathbf{\Delta }}{\boldsymbol{\varepsilon }}}_{{pm}}\left(r,\phi ,z,t\right)={b}_{{pm}}\left(\Omega ,z\right){{\boldsymbol{\mu }}}_{{pm}}\left(r,\phi \right)\exp \left({jKz}-j\Omega t\right)+c.c$$

where the elements of the transverse dependence tensor ${{\boldsymbol{\mu }}}_{{pm}}$ are of the form^1–5,41^

19 $$\left(\begin{array}{c}{\mu }_{{rr},{pm}}\left(r,\phi \right)\\ {\mu }_{\phi \phi ,{pm}}\left(r,\phi \right)\\ {\mu }_{r\phi ,{pm}}\left(r,\phi \right)\end{array}\right)=-{n}_{0}^{4}\left(\begin{array}{ccc}{p}_{11} & {p}_{12} & 0\\ {p}_{12} & {p}_{11} & 0\\ 0 & 0 & {p}_{44}\end{array}\right)\left(\begin{array}{c}{s}_{{rr},{pm}}\left(r,\phi \right)\\ {s}_{\phi \phi ,{pm}}\left(r,\phi \right)\\ 2{s}_{r\phi ,{pm}}\left(r,\phi \right)\end{array}\right)$$

The perturbations to the dielectric tensor propagate along the fiber as a wave of frequency $\Omega$ and wavenumber $K$. The perturbations scale with the modal displacement magnitude ${b}_{{pm}}\left(\Omega ,z\right)$, and hence also with the beating power between the two stimulating optical fields, $P\left(\Omega ,z\right)$ [1-5].

The acoustically induced dielectric perturbation may couple light between a pair of optical fields, ${\vec{E}}_{\mathrm{3,4}}$, propagating in optical modes 3 and 4:

20 $${\vec{E}}_{3,4}\left(r,\phi ,z,t\right)={A}_{3,4}\left(z\right){\vec{E}}_{T,3,4}(r,\phi )\exp \left(j{\beta }_{3,4}z-j{\omega }_{3,4}t\right)+c.c$$

Here ${A}_{\mathrm{3,4}}$ are the scalar complex magnitudes of the two fields [V], ${\beta }_{\mathrm{3,4}}$ are their propagation constants in their respective modes, ${\vec{E}}_{T,\mathrm{3,4}}$ are the corresponding normalized transverse profiles of the two modes, and ${\omega }_{\mathrm{3,4}}$ are their respective optical frequencies. Effective coupling requires matching in frequency and wavenumber between the pair of optical fields and the photoelastic perturbations: The difference in frequencies ${\omega }_{3}-{\omega }_{4}$ should equal the acoustic frequency $\pm \Omega$. The difference in wavenumbers ${\beta }_{3}-{\beta }_{4}$ must equal $\pm K$. In intra-modal processes, where all four optical waves involved propagate in the same mode 1, fulfillment of the frequency requirement guarantees that the wavenumbers condition is met as well. Since ${\beta }_{i}={n}_{{\rm{eff}},1}{\omega }_{i}/c$, $i=1\ldots 4$, whenever ${\omega }_{3}-{\omega }_{4}=\pm \Omega =\pm \left({\omega }_{1}-{\omega }_{2}\right)$, we automatically obtain also ${\beta }_{3}-{\beta }_{4}=\pm \left({\beta }_{1}-{\beta }_{2}\right)=\pm K$. This property holds as long as chromatic dispersion remains negligible. It suggests that intra-modal forward Brillouin scattering would couple a third, input optical signal wave with both its sidebands, spectrally detuned by $\pm \Omega$, regardless of its optical frequency. The coupling may take the form of phase modulation, polarization rotation, or both, depending on the choice of acoustic mode and the state of polarization of the optical signal wave^1–5,39^.

The requirement for wavenumber matching is markedly different in the case of inter-modal scattering, in which the acoustic wave is stimulated by optical fields in distinct spatial modes 1 and 2. Let us denote the optical frequencies ${\omega }_{\mathrm{3,4}}$ as ${\omega }_{{\rm{s}}}\pm \frac{1}{2}\Omega$, where ${\omega }_{{\rm{s}}}$ is their average. Wavenumber matching in photoelastic coupling between ${\vec{E}}_{\mathrm{3,4}}$ is reached when $\left({n}_{{\rm{eff}},3}-{n}_{{\rm{eff}},4}\right){\omega }_{{\rm{s}}}/c=\pm \left({n}_{{\rm{eff}},1}-{n}_{{\rm{eff}},2}\right){\omega }_{{\rm{p}}}/c=\pm K$. Here ${n}_{{\rm{eff}},3}$ and ${n}_{{\rm{eff}},4}$ denote the effective indices in modes 3 and 4, respectively. Wavenumber matching for photoelastic scattering in the inter-modal process is guaranteed between the two initial stimulating waves ${\vec{E}}_{\mathrm{1,2}}$, as ${\omega }_{{\rm{s}}}={\omega }_{{\rm{p}}}$, ${n}_{{\rm{eff}},3}={n}_{{\rm{eff}},1}$ and ${n}_{{\rm{eff}},4}={n}_{{\rm{eff}},2}$. In that case the forward Brillouin interaction is in the stimulated regime, with the same pair of optical waves used to both generate the acoustic wave and to monitor its induced scattering of light. Forward stimulated Brillouin scattering results in the amplification of the lower-frequency optical field, at the expense of the higher-frequency one^1–5^. If the pair of modes 3 and 4 differs from the pair of modes 1 and 2, the wavenumbers for photoelastic coupling between ${\vec{E}}_{\mathrm{3,4}}$ might only be matched for specific frequencies ${\omega }_{{\rm{s}}}$, if at all.

The photoelastic coupling between a pair of optical waves and a given acoustic mode scales with the three-way overlap integral between the transverse profiles of the dielectric perturbations and the two optical modes involved^1–5^:

21 $$\begin{array}{l}{Q}_{3,4,{pm}}^{({PE})}=\mathop{\int }\limits_{0}^{{2\pi }}\mathop{\int }\limits_{0}^{{b}}\left[{\mu }_{{rr},{pm}}{E}_{r,3}{E}_{r,4}+{\mu }_{\phi \phi ,{pm}}{E}_{\phi ,3}{E}_{\phi ,4}\right.\\\qquad\qquad\left.+\,{\mu }_{r\phi ,{pm}}\left({E}_{r,3}{E}_{\phi ,4}+{E}_{\phi ,3}{E}_{r,4}\right)\right]{rdrd}\phi\end{array}$$

Here ${E}_{r,3}$ and ${E}_{r,4}$ denote the radial components of ${\vec{E}}_{T,\mathrm{3,4}}$, respectively, and ${E}_{\phi ,3}$, ${E}_{\phi ,4}$ are the corresponding azimuthal components. One may show that when optical modes 1 and 3 are the same, and so are modes 2 and 4, the overlap integrals of electro-strictive stimulation and photoelastic coupling (Eqs. (13) and (21)) become equal: ${Q}_{\mathrm{1,2},{pm}}^{({ES})}={Q}_{\mathrm{1,2},{pm}}^{({PE})}$ ([41], see Appendix). We denote that overlap integral below as ${Q}_{\mathrm{1,2},{pm}}$. The spatial overlap vanishes unless the azimuthal order $p$ of the acoustic mode equals ${l}_{3}\pm {l}_{4}$, where ${l}_{\mathrm{3,4}}$ are the azimuthal orders of optical modes 3 and 4, respectively.

The forward Brillouin scattering gain coefficient ${\gamma }_{\mathrm{1,2},{\it pm}}\left(\Omega \right)$, between a pair of optical modes 1 and 2 and an acoustic mode ${{TR}}_{{pm}}$, is given by^5,45^

22 $${\gamma }_{1,2,{pm}}\left(\Omega \right)=j\frac{{k}_{0}{Q}_{1,2,{pm}}^{2}}{8{n}_{0}^{2}c{\rho }_{0}{\Gamma }_{{pm}}{\Omega }_{{pm}}^{\left({\rm{R}}\right)}}\frac{1}{1-2j\frac{\Omega -{\Omega }_{{pm}}^{\left({\rm{R}}\right)}}{{\Gamma }_{{pm}}}}=j{\gamma }_{1,2,{pm}}^{\left(0\right)}\frac{1}{1-2j\frac{\Omega -{\Omega }_{{pm}}^{\left({\rm{R}}\right)}}{{\Gamma }_{{pm}}}}$$

Here ${k}_{0}$ is the vacuum wavenumber at optical frequency $\tt {\omega }_{p}$, ${\rho }_{0}$ is the density of silica, and ${\Gamma }_{{pm}}$ denotes the linewidth (or decay rate) of mode ${{TR}}_{{pm}}$. That linewidth depends on the mechanical impedance of media outside the cladding, and its monitoring provides the basis for forward Brillouin fiber sensing^9^. For bare fibers in air, the linewidths are determined by acoustic dissipation in silica and by inhomogeneities in the cladding radius, and scale quadratically with acoustic frequency^46^. Typical values in bare standard single-mode fibers range between tens to hundreds of kHz.

The frequency ${\Omega }_{{pm}}^{\left({\rm{R}}\right)}$ of maximum forward Brillouin interaction is given by Eq. (11):

23 $${\Omega }_{{pm}}^{\left({\rm{R}}\right)}\approx {\Omega }_{{pm}}+\frac{{v}_{L,S}^{2}}{2{\Omega }_{{pm}}}{K}^{2}\approx {\Omega }_{{pm}}+\frac{{k}_{0}^{2}{v}_{L,S}^{2}}{2{\Omega }_{{pm}}}{\left({n}_{{\rm{eff}},1}-{n}_{{\rm{eff}},2}\right)}^{2}$$

The latter approximate equality in Eq. (23) refers to inter-modal scattering, in which the detuning from cut-off is inversely proportional to the acoustic frequency. The resonance frequency ${\Omega }_{{pm}}^{\left(R\right)}$ practically reduces to the cut-off frequency ${\Omega }_{{pm}}$ for intra-modal forward Brillouin scattering processes. The velocities ${v}_{L,S}$ apply to acoustic modes that are dominated by their dilatational or shear components, respectively.

The units of the gain coefficient ${\gamma }_{\mathrm{1,2},{\it pm}}\left(\Omega \right)$ are W^−1^ × m^−1^. It takes up its maximum magnitude on resonance:

24 $${\gamma }_{1,2,{pm}}^{\left(0\right)}=\frac{{k}_{0}}{8{n}_{0}^{2}c{\rho }_{0}}\frac{{Q}_{1,2,{pm}}^{2}}{{\Gamma }_{{pm}}{\Omega }_{{pm}}^{\left({\rm{R}}\right)}}$$

Typical ${\gamma }_{\mathrm{1,1,0}m}^{\left(0\right)}$ values for radial acoustic modes in standard, bare single-mode fibers reach the order of 10 W^−1^ × m^−1^ [5]. These values are an order of magnitude weaker than those of backward Brillouin scattering in the same fiber, in which the acoustic waves are confined to the core in larger overlap with the optical mode^6–8^. Calculations of forward Brillouin scattering spectra for a specific few-mode fiber used in this work are presented next.

### Numerical calculations of forward Brillouin scattering spectra in a few-mode fiber

The forward Brillouin scattering gain coefficients were calculated for the parameters of the few-mode fiber used in experiments (see the section Experimental Results). The fiber has a standard cladding of pure silica with radius $b$ of 62.8 µm and refractive index ${n}_{2}$ of 1.445 RIU. The uniform core of the fiber has a radius $a$ of 4.8 µm, with a refractive index ${n}_{1}$ = 1.461 RIU. The $V$ parameter of the fiber at a vacuum wavelength of 1550 nm is 4.196, and it supports the $L{P}_{01}$ and $L{P}_{02}$ modes and the $L{P}_{11}$ and $L{P}_{21}$ mode groups. The mode multiplexers used in our experiments supported the selective coupling of light to the $L{P}_{01}$ mode, $L{P}_{02}$ mode, and $L{P}_{11}$ group only, hence the $L{P}_{21}$ mode group was not considered further. The effective indices for the $L{P}_{01}$ mode, $L{P}_{02}$ mode, and $L{P}_{11}$ mode group are 1.458, 1.446, and 1.453 RIU, respectively. The differences between the effective indices of the three constituent modes of the $L{P}_{11}$ group are below 5 × 10^−^⁵ RIU. In the following calculations, the fiber was assumed to be uncoated with air outside the cladding. The modal linewidths ${\Gamma }_{{pm}}$ were fitted based on experiments^5^ (see the section Experimental Results).

**Fig. 1:**
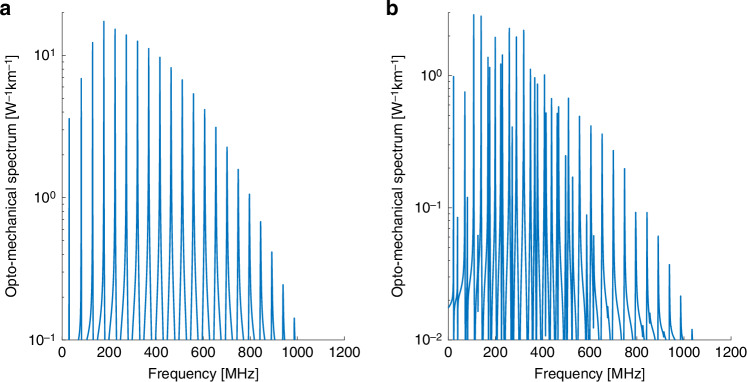
Calculated opto-mechanical spectra $\left|\boldsymbol\gamma \left(\boldsymbol\Omega /\boldsymbol2\mathbf\pi \right)\right|$ in a few-mode fiber. **a** Intra-modal forward Brillouin scattering between two optical waves in the fundamental $L{P}_{01}$ mode, through radial acoustic modes ${R}_{0m}$. **b** Same as panel **a**, with torsional-radial acoustic modes of two-fold azimuthal symmetry ${{TR}}_{2m}$

Figure 1a presents the calculated opto-mechanical spectrum $\left|{\gamma }_{{LP}01,{LP}\mathrm{01,0}\it m}\left(\Omega \right)\right|$ of intra-modal forward Brillouin scattering between two optical waves in the fundamental $L{P}_{01}$ mode, through the radial acoustic modes ${R}_{0m}$. Figure 1b shows the corresponding spectrum $|{\gamma }_{{LP}01,{LP}\mathrm{01,2}\it m}\left(\Omega \right)|$ for the same optical mode and the ${{TR}}_{2m}$ acoustic modes. Both spectra are very similar to those of the corresponding processes in standard single-mode fibers^1–5^. Forward Brillouin scattering through the ${R}_{0m}$ modes is stronger, with ${\gamma }_{{LP}01,{LP}\mathrm{01,0}\it m}^{\left(0\right)}$ reaching 20 W^−1^ × km^−1^ at frequencies near 300 MHz, compared with maximum values of 2.5–3 W^−1^ × km^−1^ for the ${{TR}}_{2m}$ modes. Similar values have been calculated and observed for single-mode fibers^39^. The peak magnitudes ${\gamma }_{{LP}01,{LP}\mathrm{01,0}\it m}^{\left(0\right)}$ decrease monotonously beyond 300 MHz frequency. The radial modes are purely dilatational, with regular spacing between their resonance frequencies (Fig. 1a). The spectrum of Fig. 1b consists of both predominantly dilatational and shear-like ${{TR}}_{2m}$ acoustic modes, with overall irregular spacing between peaks^17,18,44^. The $|{\gamma }_{{LP}01,{LP}\mathrm{01,2}\it m}\left(\Omega \right)|$ spectrum also decreases considerably beyond acoustic frequencies of 500 MHz. At higher frequencies, the radial dependence of the material displacement profile ${\vec{u}}_{{pm}}$ changes sign within the spatial extent of the fundamental optical mode, and the spatial overlap integral largely cancels out.

**Fig. 2:**
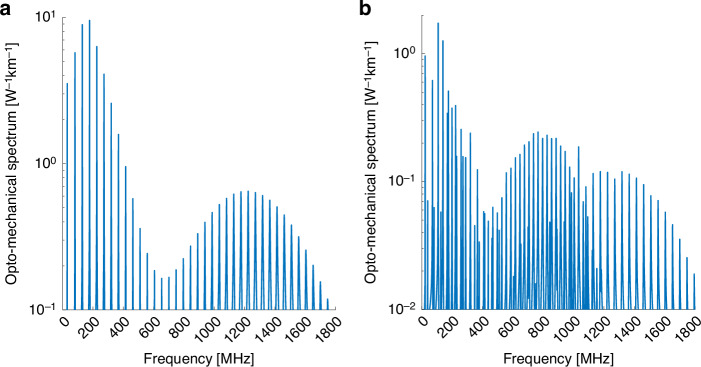
Calculated opto-mechanical spectra $\left|\boldsymbol\gamma \left(\boldsymbol\Omega /\boldsymbol2\mathbf\pi \right)\right|$ in a few-mode fiber. **a** Intra-modal forward Brillouin scattering between two optical waves in the $L{P}_{02}$ mode, through radial acoustic modes ${R}_{0m}$. **b** Same as panel **a**, with torsional-radial acoustic modes of two-fold azimuthal symmetry ${{TR}}_{2m}$

Figure 2 presents the opto-mechanical spectra of intra-modal forward Brillouin scattering in the $L{P}_{02}$ mode. ${R}_{0m}$ and ${{TR}}_{2m}$ acoustic modes are considered in Fig. 2a, b, respectively. The peaks of the radial modes spectrum $|{\gamma }_{{LP}02,{LP}\mathrm{02,0}\it m}\left(\Omega \right)|$ reach a maximum at 300 MHz and decrease by nearly two orders of magnitude towards 600 MHz. The peak magnitudes ${\gamma }_{{LP}02,{LP}\mathrm{02,0}\it m}^{\left(0\right)}$ increase again towards acoustic frequencies of 1.2 GHz. Compared with the fundamental $L{P}_{01}$ mode, the radial profiles of the displacement vectors of a high-frequency acoustic modes match better with the higher-order $L{P}_{02}$ mode, leading to more efficient Brillouin stimulation. The $|{\gamma }_{{LP}02,{LP}\mathrm{02,2}\it m}\left(\Omega \right)|$ spectrum of Fig. 2b consists again of dilatational as well as shear ${{TR}}_{2m}$ modes. In the 500–900 MHz range, the spectrum is dominated by the shear modes, identified by the closer spacing ${v}_{S}/\left(2b\right)$ between adjacent peaks. In that frequency range, the radial profiles of the shear modes better match with those of the optical mode $L{P}_{02}$. The dilatational modes, noted by a larger frequency spacing of approximately ${v}_{L}/\left(2b\right)$, become dominant beyond 1 GHz frequency.

**Fig. 3:**
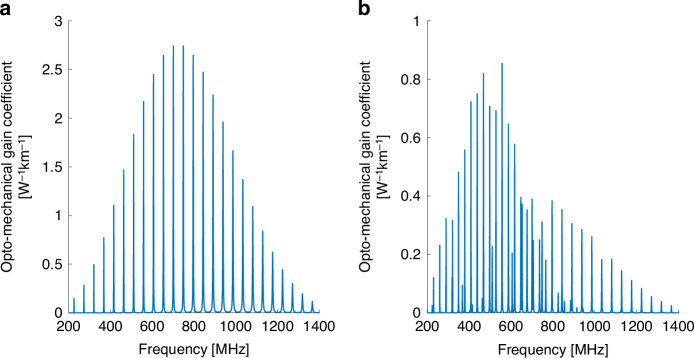
Calculated opto-mechanical gain coefficients ${\mathbf{Im}}\left\{\boldsymbol\gamma \left(\boldsymbol\Omega /\boldsymbol2\mathbf\pi \right)\right\}$ in a few-mode fiber. **a** Inter-modal forward Brillouin scattering between one optical wave in the $L{P}_{01}$ mode and another in the $L{P}_{02}$ mode, through radial acoustic modes ${R}_{0m}$. **b** Same as panel **a**, with torsional-radial acoustic modes of two-fold azimuthal symmetry ${{TR}}_{2m}$

Figure 3 shows the calculated spectra of inter-modal forward Brillouin scattering between the $L{P}_{01}$ and $L{P}_{02}$ modes. In this figure, the imaginary part of the gain coefficient ${\rm{Im}}\{{\gamma }_{{LP}01,{LP}02,{pm}}\left(\Omega \right)\}$ is plotted, rather than its absolute value as in Figs. 1 and 2, since the experimental procedure for the characterization of inter-modal scattering processes measures the imaginary part directly^5^, (see Methods Section). Here too, only the ${R}_{0m}$ and ${{TR}}_{2m}$ acoustic modes may be stimulated. Figure 3a presents the ${\rm{Im}}\{{\gamma }_{{LP}01,{LP}\mathrm{02,0}\it m}\left(\Omega \right)\}$ spectrum, and ${\rm{Im}}\{{\gamma }_{{LP}01,{LP}\mathrm{02,2}\it m}\left(\Omega \right)\}$ is plotted in Fig. 3b. Both spectra differ from those of the intra-modal scattering processes through the same acoustic modes, as shown in Fig. 1 and Fig. 2. The inter-modal scattering spectrum through the ${{TR}}_{2m}$ modes is dominated by shear modes up to 700 MHz frequency and by dilatational modes above that frequency.

**Fig. 4:**
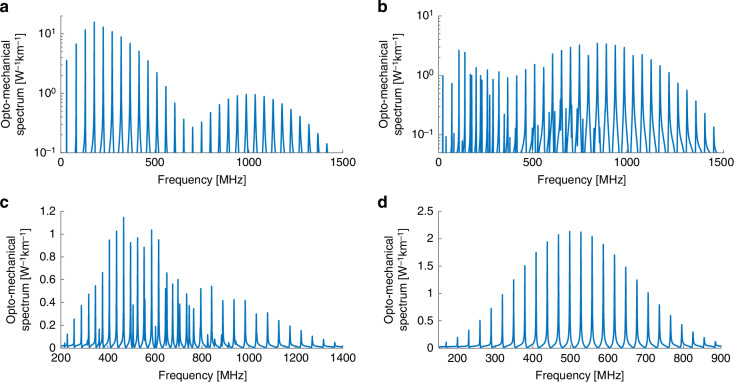
Calculated opto-mechanical spectra $\left|\boldsymbol\gamma \left(\boldsymbol\Omega /\boldsymbol2\mathbf\pi \right)\right|$ in a few-mode fiber. **a** Forward Brillouin scattering between optical waves within the $L{P}_{11}$ mode group, through radial acoustic modes ${R}_{0m}$. **b** Same as panel **a**, with torsional-radial acoustic modes of two-fold azimuthal symmetry ${{TR}}_{2m}$. **c** Same as panel **a**, with torsional-radial acoustic modes of four-fold azimuthal symmetry ${{TR}}_{4m}$. **d** Same as panel **a**, with purely torsional acoustic modes ${T}_{0m}$

Figure 4 presents calculated spectra of forward Brillouin scattering interactions driven by optical fields in the $L{P}_{11}$ mode group. The group consists of the $T{E}_{01}$, $T{M}_{01}$ and $H{E}_{21}$ exact modal solutions. The experimental setup only allows for the coupling of light to all three modes together, and it does not separate between them. Figure 4a presents the $|{\gamma }_{{LP}11,{LP}\mathrm{11,0}\it m}\left(\Omega \right)|$ forward Brillouin scattering spectrum through the radial ${R}_{0m}$ acoustic modes. The spectrum includes contributions of intra-modal scattering in each of the three modes within the $L{P}_{11}$ mode group. The radio frequency phases of the three contributions may vary. In the example shown here, the mean value of the three terms is presented. Differences between the acoustic resonance frequencies of the three contributions are much smaller than the modal linewidths and cannot be observed. The spectrum extends towards higher frequencies than those of the corresponding process in the fundamental $L{P}_{01}$ mode (Fig. 1a). The frequencies range is similar to that of the intra-modal scattering in the $L{P}_{02}$ mode (Fig. 2a). The forward Brillouin scattering spectrum $|{\gamma }_{{LP}11,{LP}\mathrm{11,2}\it m}\left(\Omega \right)|$ for ${{TR}}_{2m}$ acoustic modes is shown in Fig. 4b. The spectrum differs from those of ${{TR}}_{2m}$ stimulated by other optical modes combinations, as presented earlier. It is dominated by dilatational modes above acoustic frequencies of 800 MHz.

Optical waves within the $L{P}_{11}$ modes group can stimulate additional classes of acoustic modes, beyond the ${R}_{0m}$ and ${{TR}}_{2m}$ groups discussed thus far. Figure 4c shows the spectrum $|{\gamma }_{{LP}11,{LP}\mathrm{11,4}\it m}\left(\Omega \right)|$ of forward Brillouin scattering through the four-fold symmetric ${{TR}}_{4m}$ acoustic modes. These modes can be driven by two pump fields in the $H{E}_{21}$ optical mode within the $L{P}_{11}$ group. This class of acoustic modes cannot be observed in forward Brillouin scattering over single-mode fibers. Shear modes within the ${{TR}}_{4m}$ category dominate the spectrum up to 700 MHz, giving way to dilatational modes at higher frequencies. The cut-off frequencies ${\Omega }_{4m}$ of the ${{TR}}_{4m}$ modes are similar to those of the ${{TR}}_{2m}$ modes. However, the ${\Omega }_{4m}$ frequencies are consistently lower than the corresponding ${\Omega }_{2m}$ values by 2π × 1-2 MHz. These offsets are used to identify peaks associated with the ${{TR}}_{4m}$ modes in experimental measurements (see the section Experimental Results).

Inter-modal forward Brillouin scattering between one optical field in the $T{E}_{01}$ mode and another in the $T{M}_{01}$ mode within the $L{P}_{11}$ group can stimulate purely torsional ${T}_{0m}$ acoustic modes as well. The calculated spectrum is shown in Fig. 4d. The process couples optical power between the $T{E}_{01}$ and $T{M}_{01}$ components within the $L{P}_{11}$ mode group. The cut-off frequencies of the ${T}_{0m}$ modes are higher than those of adjacent ${{TR}}_{2m}$ modes by 2π × 1-2 MHz. The peak magnitudes of the opto-mechanical stimulation of ${T}_{0m}$ modes are highest near 500 MHz frequency. Material displacement in the ${T}_{0m}$ modes includes shear wave motion only.

**Fig. 5:**
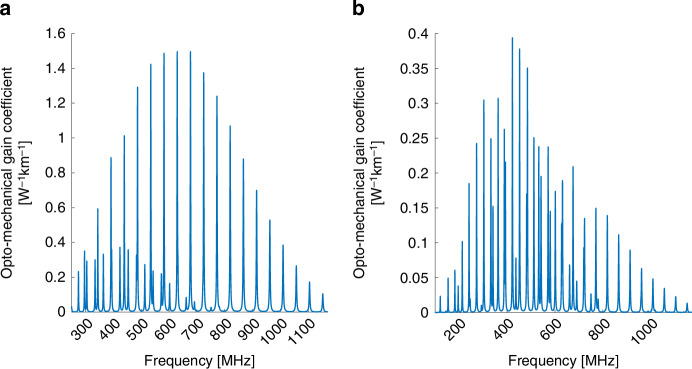
Calculated opto-mechanical gain coefficients ${\mathbf{Im}}\left\{\boldsymbol\gamma \left(\boldsymbol\Omega /\boldsymbol2\mathbf\pi \right)\right\}$ in a few-mode fiber. **a** Inter-modal forward Brillouin scattering between one optical wave in the $L{P}_{01}$ mode and another in the $L{P}_{11}$ mode group, through torsional-radial acoustic modes $T{R}_{1m}$ of first-order azimuthal symmetry. **b** Same as panel **a**, with torsional-radial acoustic modes of three-fold azimuthal symmetry ${{TR}}_{3m}$

Lastly, Fig. 5 shows the calculated spectra of inter-modal forward Brillouin scattering between one optical wave in the fundamental $L{P}_{01}$ mode and another in the $L{P}_{11}$ modes group. The process involves the stimulation of the ${{TR}}_{1m}$ acoustic modes of first-order azimuthal symmetry (panel a), and the three-fold symmetric ${{TR}}_{3m}$ modes (Fig. 5b). Both classes of modes are inaccessible to forward Brillouin scattering in single-mode fibers. The peak magnitudes of ${\rm{Im}}\{{\gamma }_{{LP}01,{LP}\mathrm{11,1}\it m}\left(\Omega \right)\},$ corresponding to the ${{TR}}_{1m}$ modes, are four times stronger than those of ${\rm{Im}}\{{\gamma }_{{LP}01,{LP}\mathrm{11,3}\it m}\left(\Omega \right)\}$, representing the ${{TR}}_{3m}$ modes. The spectrum of ${{TR}}_{1m}$ modes is dominated by the dilatational ones.

### Experimental results

**Fig. 6:**
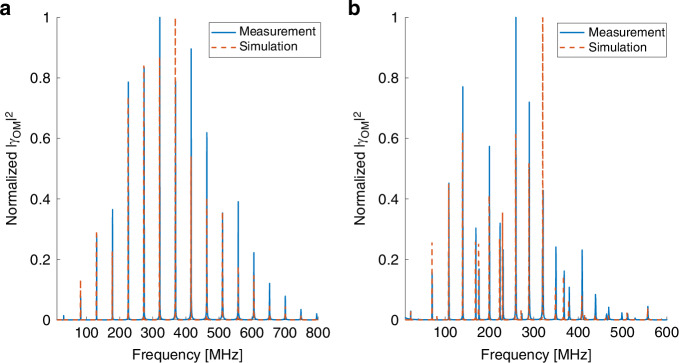
Measured (solid, blue) and calculated (dashed, red) normalized spectra ${\left|{\boldsymbol\gamma }_{\boldsymbol{LP}{\boldsymbol{01}},{\boldsymbol{LP}}{\boldsymbol{01}},{\boldsymbol{pm}}}\left(\boldsymbol\Omega \right)\right|}^{\boldsymbol{2}}$ of intra-modal forward Brillouin scattering in the fundamental ${\boldsymbol{LP}}_{\boldsymbol{01}}$ mode of a few-mode fiber. **a** Radial acoustic modes ${R}_{0m}$. **b** Torsional-radial modes. The spectrum consists of the ${{TR}}_{2m}$ modes only

Figure 6a shows the measured normalized spectrum ${|{\gamma }_{{LP}01,{LP}\mathrm{01,0}\it m}\left(\Omega \right)|}^{2}$ of intra-modal forward Brillouin scattering in the fundamental $L{P}_{01}$ mode of a few-mode fiber, through the radial acoustic modes ${R}_{0m}$. For the details of the experimental setup and protocols, see the Methods section. The fiber under test was 5 meters long, and it was stripped off its protective polymer coating to enhance forward Brillouin scattering^9^. The parameters of the fiber were the same as those used above for calculations. The corresponding calculated spectrum is plotted as well (see Fig. 1a). The agreement between experiment and calculations is very good. The spectrum is very similar to that of forward Brillouin scattering through the same acoustic modes in single-mode fibers^1–5^, as expected. The linewidths of the spectral peaks increase with frequency, from 100 kHz width for the 80 MHz peak up to about 1 MHz width at the resonance frequency of 700 MHz. The linewidths are broader than previously observed in uncoated single-mode fibers, and they scale linearly with acoustic frequency. Such scaling suggests broadening due to nonuniformity of the cladding diameter along the fiber under test^46^. Figure 6b presents the normalized measured and calculated spectra of intra-modal scattering in the fundamental optical mode through torsional-radial acoustic modes. The spectrum consists of two-fold symmetric ${{TR}}_{2m}$ acoustic modes only. Here too, the experimental results agree with calculations and with known results in single-mode fibers^1–5^. Both dilatational and shear modes are observed. Note that the forward Brillouin scattering spectra through radial and torsional-radial modes are acquired using different detection schemes (see Methods). Therefore, the comparison between spectra in absolute scales is not provided. For experimental comparison between radial and torsional-radial modes excitation, see earlier works^39^.

**Fig. 7:**
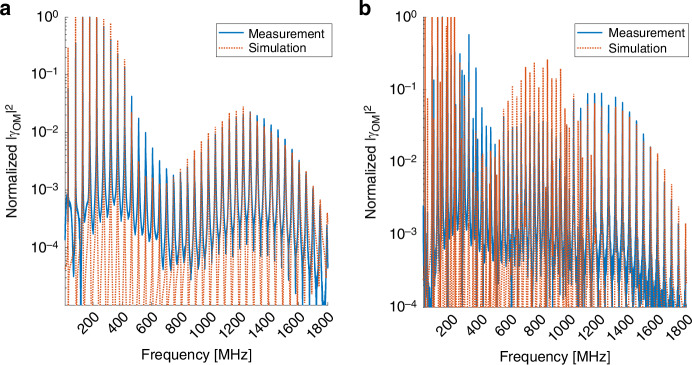
Measured (solid, blue) and calculated (dashed, red) normalized spectra ${\left|\boldsymbol{\gamma }_{\boldsymbol{LP}{\boldsymbol{02}},{\boldsymbol{LP}}{\boldsymbol{02}},{\boldsymbol{pm}}}\left(\boldsymbol\Omega \right)\right|}^{\boldsymbol{2}}$ of intra-modal forward Brillouin scattering in the ${\boldsymbol{LP}}_{\boldsymbol{02}}$ mode of a few-mode fiber. **a** Radial acoustic modes ${R}_{0m}$. **b** Torsional-radial modes. The spectrum consists of the ${{TR}}_{2m}$ modes only

Figure 7 shows the measured and calculated normalized spectra of intra-modal forward Brillouin scattering in the higher-order $L{P}_{02}$ mode. Here too, the process takes place through ${R}_{0m}$ (panel a) and ${{TR}}_{2m}$ acoustic modes (panel b). Modes of both categories are observed up to a frequency of 1.8 GHz, much higher than those of corresponding intra-modal scattering in the fundamental mode. The peak magnitudes of the dilatational ${R}_{0m}$ modes pass through a local minimum at acoustic frequencies near 800 MHz, due to poor spatial overlap with the optical mode. The ${{TR}}_{2m}$ modes spectrum at that frequency range is dominated by the shear modes, identified by their closer spectral spacing, which exhibit better spatial overlap with the optical mode. The dilatational modes dominate the ${{TR}}_{2m}$ spectrum beyond 1.1 GHz frequency. The experimental observations are in good agreement with calculations.

**Fig. 8:**
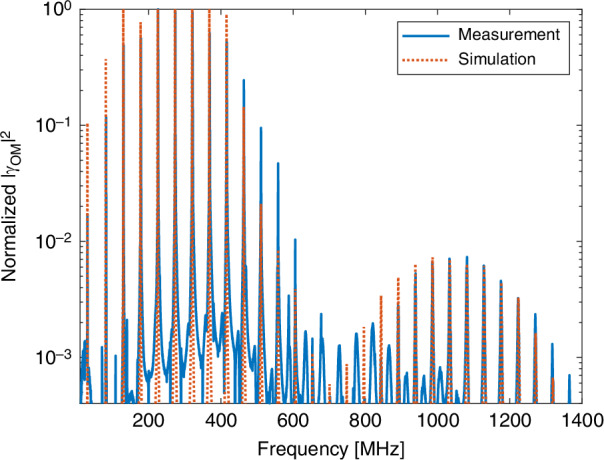
Measured (solid, blue) and calculated (dashed, red) normalized spectra ${\left|{\gamma }_{{LP}11,{LP}\mathrm{11,0}\it m}\left(\Omega \right)\right|}^{2}$ of intra-modal forward Brillouin scattering in the ${{LP}}_{11}$ modes group of a few-mode fiber, through the radial acoustic modes ${R}_{0m}$

**Fig. 9:**
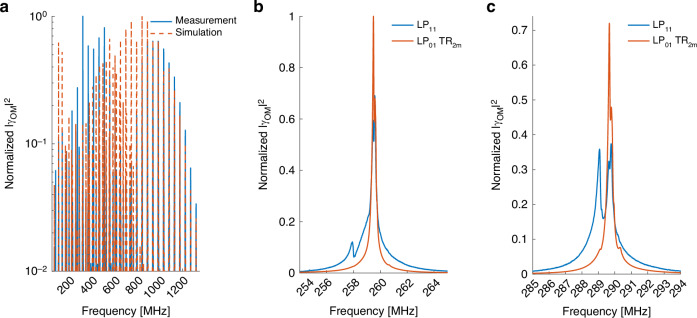
Observation of four-fold symmetric torsional-radial acoustic modes. **a** Measured (solid, blue) and calculated (dashed, red) normalized spectra of intra-modal forward Brillouin scattering in the ${{LP}}_{11}$ modes group of a few-mode fiber, through torsional-radial acoustic modes. The spectrum is dominated by the ${{TR}}_{2m}$ modes. **b**, **c** Magnified views of the experimental trace of panel (a) (blue), alongside the measured, normalized torsional-radial modes spectrum of intra-modal scattering in the ${{LP}}_{01}$ mode (red, repeated from Fig. 6b as a reference). The torsional-radial acoustic modes spectrum following stimulation through the ${{LP}}_{11}$ modes group exhibits secondary peaks, which do not appear in the corresponding ${{LP}}_{01}$ process. The frequencies of the secondary peaks are lower than those of the primary ones by 1–2 MHz. The secondary peaks represent the stimulation of ${{TR}}_{4m}$ acoustic modes. Two examples of pairs of peaks are shown in the figure, however many others were observed

Next, the normalized spectra of forward Brillouin scattering between two optical fields in the $L{P}_{11}$ group are presented. Figure 8 shows the spectrum of scattering through the radial ${R}_{0m}$ modes, ${|{\gamma }_{{LP}11,{LP}\mathrm{11,0}\it m}\left(\Omega \right)|}^{2}$. The measurements meet expectations. Figure 9a shows the corresponding spectra for torsional radial modes. The spectrum is dominated by the two-fold symmetric ${{TR}}_{2m}$ modes, and it matches well with calculations. This time, however, many of the spectral peaks corresponding to the ${{TR}}_{2m}$ modes are accompanied by adjacent secondary peaks, at frequencies that are 1–2 MHz lower. The secondary set of peaks represents the stimulation of the four-fold symmetric ${{TR}}_{4m}$ modes. The observed frequency separation matches the difference between calculated cut-off frequencies $\left({\Omega }_{2m}-{\Omega }_{4m}\right)/2\pi$. The ${{TR}}_{4m}$ acoustic modes are driven by optical fields in the ${{HE}}_{21}$ component of the ${{LP}}_{11}$ group. They do not appear in the spectra of torsional-radial modes for intra-modal forward Brillouin scattering in the ${{LP}}_{01}$ and ${{LP}}_{02}$ modes (see Fig. 9). Two examples of pairs of peaks are shown in Fig. 9b, c, and many other pairs were observed in the frequencies range of 200–400 MHz. The results signify the first observation of the ${{TR}}_{4m}$ class of modes in forward Brillouin scattering in fibers.

Calculations also predict the stimulation of purely torsional ${T}_{0m}$ acoustic modes by one field component in the ${{TE}}_{01}$ mode and another in the ${{TM}}_{01}$ mode within the $L{P}_{11}$ group (see Fig. 4d). Such stimulation would be accompanied by the coupling of optical power between the two components and may lead to polarization rotation of the combined $L{P}_{11}$ polarized field. The cut-off frequencies of the ${T}_{0m}$ acoustic modes are higher than those of adjacent ${{TR}}_{2m}$ modes by 2π × 1-2 MHz (as opposed to the cut-of frequencies of the ${{TR}}_{4m},$ which are lower than those of the ${{TR}}_{2m}$ modes by comparable offsets). We were unable to resolve the stimulation of ${T}_{0m}$ acoustic modes in our measurements. The division of the input field among the three constituent modes of the $L{P}_{11}$ group is not controlled. The magnitudes of the launched ${{TE}}_{01}$ and ${{TM}}_{01}$ field components might have been too weak, and the polarization rotation induced by the ${T}_{0m}$ modes might have been too small to be identified alongside the much stronger response of adjacent ${{TR}}_{2m}$ modes.

**Fig. 10:**
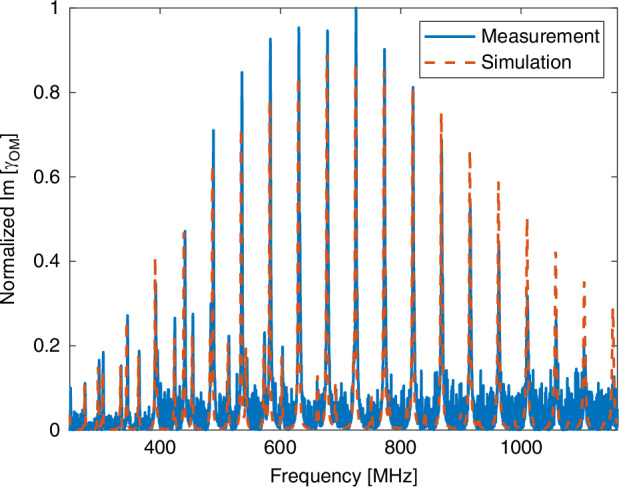
Observation of torsional radial acoustic modes of first-order azimuthal symmetry. Solid blue—measured normalized gain coefficient of inter-modal forward stimulated Brillouin scattering between one optical wave in the fundamental ${{LP}}_{{01}}$ mode and another in the ${{LP}}_{{11}}$ mode group. Dashed red—calculated normalized gain coefficient ${{Im}}\left\{{{\gamma}}_{{LP}}{{01}},{{LP}}{{11,1}}{\it m}\right\}$ of inter-modal forward Brillouin scattering between the two optical modes through the ${{TR}}_{1m}$ acoustic modes. Agreement with experiment is very good. The weaker stimulation of ${{TR}}_{3m}$ modes, suggested by the analysis, was not observed in the measurements

The measured normalized coupling coefficient ${\rm{Im}}\left\{\gamma \left(\Omega \right)\right\}$ of inter-modal forward Brillouin scattering between the $L{P}_{01}$ optical mode and the $L{P}_{11}$ mode group is presented in Fig. 10. For the experimental setup and protocols for measuring inter-modal scattering, see Methods Section. The spectrum consists of stimulated ${{TR}}_{1m}$ acoustic modes of first-order azimuthal symmetry. Forward Brillouin scattering through this set of acoustic modes is also observed for the first time in this work. Like the ${{TR}}_{4m}$ modes seen in Fig. 9, the ${{TR}}_{1m}$ set of modes cannot be stimulated in single mode fibers. The cut-off frequencies ${\Omega }_{1m}$ of these modes differ from ${\Omega }_{0m}$ or ${\Omega }_{2m}$ by tens of MHz, hence the observed peaks are distinct. The ${R}_{0m}$ and ${{TR}}_{2m}$ acoustic modes were not stimulated in this experiment, as expected. The spectrum is dominated by dilatational modes, although shear modes are observed as well at acoustic frequencies below 600 MHz.

Our analysis suggests that the inter-modal scattering process would also excite the ${{TR}}_{3m}$ class of acoustic modes. However, the opto-mechanical coefficients for the stimulation of these modes are considerably smaller than those of the ${{TR}}_{1m}$ modes (see Fig. 5), and the cut-off frequencies ${\Omega }_{1m}$ and ${\Omega }_{3m}$ of the two classes differ by only 2π × 1–2 MHz. We were unable to resolve the stimulation of ${{TR}}_{3m}$ modes in our experiments, and their study remains for future work.

**Fig. 11:**
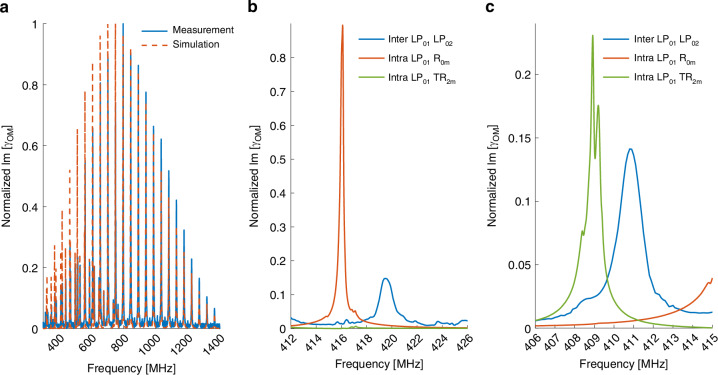
Stimulation of acoustic modes above their cut-off frequencies. **a** Solid blue—measured normalized gain coefficient of inter-modal forward stimulated Brillouin scattering between one optical wave in the fundamental ${{LP}}_{01}$ mode and another in the ${{LP}}_{02}$ mode. Dashed red—calculated normalized gain coefficient of the inter-modal scattering process through the ${R}_{0m}$ and ${{TR}}_{2m}$ acoustic modes. Agreement with experiment is very good. **b**, **c** Magnified view of the measured normalized inter-modal scattering spectrum (blue), alongside the measured normalized spectra of intra-modal scattering in the fundamental ${{LP}}_{01}$ mode. In both panels, ${R}_{0m}$ modes are shown in red and ${{TR}}_{2m}$ modes in green. In panel **b**, the resonance frequency of a dilatational ${R}_{0m}$ mode is higher by 3.4 MHz in the intra-modal trace. The corresponding difference for the ${{TR}}_{2m}$ shear mode shown in panel **c** is 1.9 MHz

**Fig. 12:**
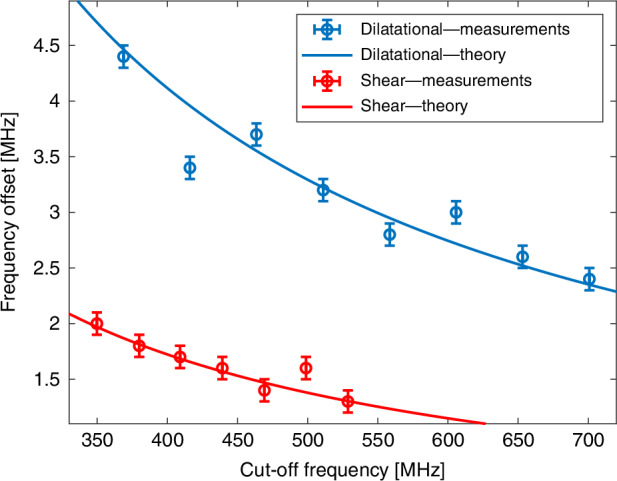
Stimulation of acoustic modes above their cut-off frequencies. Circular markers—measured spectral offsets $\Delta\Omega /2\pi$ between the resonance frequencies of inter-modal forward Brillouin scattering between the ${{LP}}_{01}$ and ${{LP}}_{02}$ modes, and the corresponding resonance frequencies of intra-modal scattering. The experimental uncertainties represent the measurement resolution of 100 kHz. Solid lines—calculated offsets according to Eq. (23). Modes dominated by their dilatational components are shown in blue, whereas those that are primarily of shear characteristics are shown in red. The observed spectral offsets agree with analysis. The offsets are inversely proportional to frequency, and they are larger for the dilatational modes due to their higher acoustic velocity

The normalized spectrum of inter-modal forward Brillouin scattering between the $L{P}_{01}$ and $L{P}_{02}$ optical modes is presented in Fig. 11a. The measured response consists of the ${R}_{0m}$ and ${{TR}}_{2m}$ acoustic modes, and it agrees well with expectations. Careful comparison between the response of Fig. 11a and the spectra of ${R}_{0m}$ and ${{TR}}_{2m}$ modes obtained through intra-modal scattering reveals a significant difference. The resonance frequencies ${\Omega }_{0m}^{\left({\rm{R}}\right)}$ and ${\Omega }_{2m}^{\left({\rm{R}}\right)}$ of the inter-modal scattering spectrum are consistently higher than those observed through intra-modal scattering. Examples are shown in Fig. 11b, c. The difference is due to the larger axial wavenumber $K$ of inter-modal electro-strictive stimulation (see earlier analysis). Figure 12 plots the difference $\Delta \Omega$ between the inter-modal and intra-modal resonance frequencies, as a function of $\Omega$. The difference is inversely proportional to $\Omega$, and it follows the prediction of Eq. (23). The difference $\Delta \Omega$ is larger for dilatational acoustic modes, due to their higher velocity.

## Discussion

This work extended the study of forward Brillouin scattering in standard, uniform-cladding fibers to the multi-optical mode regime, through analysis, calculations, and experiment. The process supports many possible combinations of intra-modal and inter-modal scattering. Unlike polarization maintaining or photonic crystal fibers, the forward Brillouin scattering spectra in the few-mode fiber are calculated analytically. Acoustic modes of arbitrary azimuthal orders can be stimulated in the few-mode fiber, as opposed to the ${R}_{0m}$ and ${{TR}}_{2m}$ modes only in single-mode fibers. The stimulation of ${{TR}}_{1m}$ and ${{TR}}_{4m}$ modes has been demonstrated experimentally.

The forward Brillouin scattering spectra of the few-mode fiber extend to higher acoustic frequencies than in standard single-mode fibers: resonance frequencies up to 1.8 GHz were observed in the measurements. Further, inter-modal stimulation in the few-mode fiber may excite acoustic modes a few MHz above their cut-off frequencies. The acoustic modes, in this case, may take up axial wavenumbers in the order of 10^4 ^rad × m^−1^. Such large wavenumbers can lead to non-reciprocal coupling of counter-propagating signal waves between spatial optical modes and to narrowband optical isolation, as shown in polarization maintaining fibers and photonic integrated circuits^38,47^. The effect of media outside the cladding on the acoustic linewidth may differ among classes of modes^44^. Addressing additional mode groups may enhance the application of forward Brillouin scattering in fiber sensing^17,18,44^. The elastic properties of media under test may also vary with acoustic frequency. The extension of forward Brillouin scattering towards higher acoustic frequencies over few-mode fibers would enable broader characterization of acoustic dispersion.

Inter-modal forward Brillouin scattering is associated with the amplification of one input optical field at the expense of another^1–5^. This amplification mechanism has been the basis for a forward Brillouin laser over polarization maintaining fiber^48^, and may support similar lasing in few-mode fiber as well. The gain bandwidth of forward Brillouin scattering is extremely narrow, only 100 kHz in bare fibers, hence forward Brillouin lasers can become extremely coherent. The boundary conditions of acoustic modes make forward Brillouin fiber lasers highly sensitive to their environment^48^. Compared with polarization maintaining fibers, the few-mode fibers provide greater freedom for the choice of modes and the design of forward Brillouin lasers.

Both the optical and acoustic waves used in this work can carry angular momenta in their orbital degree of freedom^39,40^. The forward Brillouin scattering interactions through specific choices of modes signify the exchange of orbital angular momentum quanta between the optical and mechanical domains. These interactions may potentially serve towards the manipulation of quantum states at cryogenic temperatures.

While the reported characterization of forward Brillouin scattering in the few-mode fiber under test has been rather extensive, it is by no means exhaustive. Even with the three-mode fiber available to us, there are many additional possible combinations for the allocation of input fields to specific modes. For example, two intra-modal scattering processes can be coupled through a common acoustic mode: A pair of pump waves in a common optical mode 1 would stimulate the acoustic wave through a first intra-modal process, and a signal wave in optical mode 2 would be scattered by the same acoustic wave in a second intra-modal process. We have successfully demonstrated such coupling between intra-modal forward Brillouin scattering processes in polarization-maintaining fibers^38^, and their exploration over few-mode fibers remains for future study.

In this work, we were not able to resolve the simulation of ${{TR}}_{3m}$ acoustic modes alongside the ${{TR}}_{1m}$ ones in an inter-modal forward Brillouin scattering process, even though our analysis suggests that these modes should have been observed. The difficulty may have to do with the limited signal-to-noise ratio of the inter-modal measurements protocol. The signal-to-noise ratio may be improved using longer, uncoated fibers. Another limitation stems from the small separation between the cut-off frequencies of the ${{TR}}_{1m}$ and ${{TR}}_{3m}$ modes. The spectra of the ${{TR}}_{3m}$ modes might be overshadowed by adjacent, stronger peaks due to ${{TR}}_{1m}$ modes. We also could not observe the stimulation of the ${T}_{0m}$ acoustic modes by optical fields within the ${{LP}}_{11}$ mode group. The monitoring of forward Brillouin scattering within the ${{LP}}_{11}$ group relies on polarization rotation. It is possible that the rotation associated with the coupling of light between the ${{TE}}_{01}$ and ${{TM}}_{01}$ field components within the ${{LP}}_{11}$ group was too weak to be detected. The identification of the ${T}_{0m}$ acoustic mode is also challenging due to the presence of adjacent, stronger peaks associated with the ${{TR}}_{2m}$ modes category. The stimulation of the ${T}_{0m}$ and ${{TR}}_{3m}$ will be revisited in future work.

In summary, the study of forward Brillouin scattering in standard few-mode fibers extends the fundamental understanding and formulation of the effect, reaches higher acoustic frequencies and additional modal symmetries, and may find applications in fiber sensing, fiber lasing, non-reciprocal propagation, and quantum technologies.

## Materials and methods

**Fig. 13:**
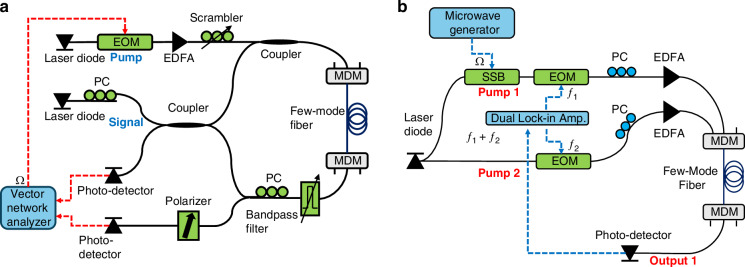
Schematic illustrations of experimental setups used in characterization of forward Brillouin scattering in a few-mode fiber. **a** Intra-modal scattering. **b** Inter-modal scattering. EOM Mach-Zehnder electro-optic intensity modulator, SSB single-sideband electro-optic modulator, EFDA erbium-doped fiber amplifier, MDM mode division multiplexer, PC polarization controller

Figure 13a illustrates the experimental setup for the characterization of intra-modal forward Brillouin scattering in a few-mode fiber^5,10,44,49^. A laser diode of 1550 nm wavelength was the source of pump optical waves used to stimulate forward Brillouin scattering in the fiber. Pump light passed through an electro-optic intensity modulator, driven by a sine wave voltage of variable radio frequency $\Omega$ from the output port of an electrical vector network analyzer. The modulator was biased at quadrature. The modulated pump wave was amplified in an erbium-doped fiber amplifier to an average optical power of 0.2–0.4 W and launched to one of the three input ports of a first mode-division multiplexer. In some of the measurements, a polarization scrambler was used in the pump branch. The magnitude of the pump power modulation ${P}_{p}\left(\Omega \right)$ was calibrated for each radio frequency.

The mode multiplexer coupled the input pump wave to either the fundamental $L{P}_{01}$ mode, the $L{P}_{02}$ mode, or the $L{P}_{11}$ mode group. The few-mode fiber under test (SK Photonics FMF) was 5 m long, and its parameters were specified in the description of numerical calculations. The fiber was stripped off its protective polymer coating to enhance forward Brillouin scattering. The coating was removed through mechanical stripping and overnight immersion in acetone for the removal of residues. The output end of the fiber under test was connected to a second mode division multiplexer which separated the $L{P}_{01}$ mode, the $L{P}_{02}$ mode, and the $L{P}_{11}$ group into three different physical output ports. An optical bandpass filter blocked the pump wave at the mode multiplexer output.

A continuous-wave signal from a second laser diode was used for monitoring the stimulated acoustic waves through photoelastic scattering. The signal wavelength was 1532 nm and its optical power was 2 mW. The signal wave was coupled with the modulated pump and launched into the same input port of the mode division multiplexer. The stimulated acoustic waves imprinted phase modulation and/or polarization rotation at radio frequency $\Omega$ on the co-propagating signal wave^5^. The optical bandpass filter at the fiber under test output was adjusted to transmit the signal wave.

The output signal was analyzed through two detection channels. In one channel, the output signal was connected through a directional coupler to form a Sagnac interferometer loop^49^. Photoelastic phase modulation of the signal wave was converted to an intensity reading at the loop output^49^. Polarization controllers were used to maximize the output intensity modulation^49^. The output signal wave was detected by a photo-receiver of 1.6 GHz bandwidth, and the obtained voltage was connected to the input port of the electrical vector network analyzer. The analyzer measured the transfer function $S\left(\Omega \right)$ of radio frequency voltage between the modulation of the optical pump wave and that of the detected signal wave. Traces were acquired with frequency steps of 10–20 kHz and an intermediate frequency bandwidth of 100 kHz and were averaged over 200 repetitions.

The frequency response of the forward Brillouin scattering process under test was estimated by the ratio $H\left(\Omega \right)=S\left(\Omega \right)/{P}_{p}\left(\Omega \right)$ [5,49]. The polarization of the pump wave was scrambled during data acquisition at hundreds of kHz rates. In that manner, the contributions of all TR modes to the photoelastic modulation were canceled out of the collected data^39^. The measured response $H\left(\Omega \right)$ was affected by the intra-modal forward Brillouin scattering through the radial modes ${R}_{0m}$ only.

The signal wave at the output of the optical bandpass filter was split into a second detection channel which was based on a polarizer. A polarization controller was used to align the signal state of polarization to 50% transmission of optical power to the polarizer output. Photoelastic polarization rotation of the signal wave was converted to intensity modulation at the polarizer output^1,10,44^. Polarization rotation takes place through torsional-radial modes of all orders, but not through radial modes^39^. The output signal was routed to an identical photo-receiver and the detected voltage was analyzed by the vector network analyzer using the same settings. The transfer function $H\left(\Omega \right)=S\left(\Omega \right)/{P}_{p}\left(\Omega \right)$ of radio-frequency voltage in this case is related to the intra-modal forward Brillouin scattering through all ${{TR}}_{{pm}}$ modes, of all azimuthal orders^39^. The polarization of the pump wave was not scrambled when using this detection channel. Since the radial and torsional-radial modes spectra are detected through different channels, the direct comparison between the absolute magnitudes of their excitation is not supported by the setup.

In addition to forward Brillouin scattering, the pump wave also induced phase modulation and polarization rotation of the signal via the Kerr effect^50^. The response of the Kerr effect is that of an immediate impulse, whereas that of photoelastic modulation extends over microseconds scale. The contribution of the Kerr effect may be effectively removed from the measurements using time gating, which eliminates the first few nanoseconds of the response^49^. Such gating is often performed by a synchronized optical switch within the experimental testbed^49^. Alternatively, when the output signal is acquired in the time domain, gating can be implemented through offline processing^9,50^. In this work, the forward Brillouin scattering processes were monitored in the frequency domain, and time gating could not be implemented directly. Instead, the inverse-Fourier transform of the complex-valued $H\left(\Omega \right)$ was calculated offline to obtain the corresponding time-domain impulse response $h\left(t\right)$. The impulse response was then gated to remove the contribution of the Kerr effect, and the Fourier transform $\widetilde{H}\left(\Omega \right)$ of the gated $\widetilde{h}\left(t\right)$ was calculated for further data analysis. The obtained $\widetilde{H}\left(\Omega \right)$ is proportional to the intra-modal forward Brillouin scattering coefficient ${\gamma }_{\mathrm{1,1},{\it pm}}\left(\Omega \right)$, through either the radial or the torsional-radial modes.

Figure 13b presents a schematic illustration of the setup for characterization of inter-modal forward Brillouin scattering in the same few-mode fiber^38,51^. Light from a laser diode source of 1550 nm wavelength was split into two paths. Light in the upper branch passed through a single-sideband electro-optic modulator, driven by voltage of variable radio frequency $\Omega$ from the output port of a microwave generator. The optical frequency of the upper branch wave was thereby offset by $\Omega$. The light wave was then intensity-modulated in an electro-optic Mach-Zehnder modulator, driven by a voltage of frequency ${f}_{1}$ = 200 kHz from one output port of a dual-channel lock-in amplifier. The modulated wave was amplified to an average optical power of 0.1 W and launched to the few-mode fiber through one input port of the mode-division multiplexer.

The optical wave at the lower branch was intensity modulated at frequency ${f}_{2}$ = 503 kHz in a second electro-optic Mach-Zehnder modulator, driven by voltage from a second output port of the lock-in amplifier. The lower branch wave was amplified to 0.5 W average power and launched through the fiber under test through a second, different port of the mode division multiplexer. Inter-modal forward Brillouin scattering may lead to the coupling of optical power between the two input waves, depending on their frequency offset $\Omega$. Such coupling manifests in intensity modulation of both waves at frequencies ${f}_{1}\pm {f}_{2}$ [38,51].

Light from one of the output ports of the second mode division multiplexer, located at the far end of the fiber under test, was detected using a photo-receiver of 20 GHz bandwidth. The detected voltage was analyzed at the input port of the lock-in amplifier, and the magnitude of the ${f}_{1}+{f}_{2}$ frequency component was monitored. That component is proportional to the coupling coefficient of inter-modal forward Brillouin scattering, ${\rm{Im}}\left\{\gamma \left(\Omega \right)\right\}$ [38]. The coupling of power may take place through all acoustic modes, radial and torsional-radial alike. Note that the Kerr effect does not induce coupling of optical power between the two fields, and its removal was not required as part of this protocol.

## Data Availability

The data that supports the findings of this study are available from the corresponding author upon reasonable request.

## Appendix A

**Spatial overlap integrals of electro-strictive stimulation and photoelastic scattering**

In this Appendix, we show that the two overlap integrals used in our analysis, that of electro-strictive stimulation and that of photoelastic scattering, are identical. The derivation is detailed in Cartesian coordinates for convenience, but it holds for any choice of coordinates system.

Let us denote the normalized transvers profiles of optical modes 1 and 2 as ${\vec{E}}_{T\mathrm{1,2}}\left(x,y\right)$ [m^−1^], where $x$ and $y$ are the Cartesian coordinates in the transverse plane. Each profile consists of two components:

A1 $${\vec{E}}_{T1,2}\left(x,y\right)={E}_{x1,2}\left(x,y\right)\hat{{\boldsymbol{x}}}{\boldsymbol{+}}{E}_{y1,2}\left(x,y\right)\hat{{\boldsymbol{y}}}$$

Here $\hat{{\boldsymbol{x}}}$ and $\hat{{\boldsymbol{y}}}$ denote the unit vectors in the $x$ and $y$ directions, respectively. We define next the tensor ${{\boldsymbol{I}}}_{\mathrm{1,2}}$ of cross-products between normalized field components. The elements of this tensor are

A2 $${I}_{{ij},1,2}={E}_{i1}{E}_{j2}$$

where $i,j$ may equal $x$ or $y$. Next, we may express the normalized transverse dependence of the electro-strictive stress tensor as follows^41^ (see Eq. (5)):

A3 $${\widetilde{{\boldsymbol{\sigma }}}}_{T1,2}=-{n}_{0}^{4}{{\boldsymbol{p}}}_{T}:{{\boldsymbol{I}}}_{1,2}$$

Here, the $:$ symbol denotes the tensor product, and ${{\boldsymbol{p}}}_{T}$ is the transverse sub-set of the fourth-rank photo-elastic tensor ${\boldsymbol{p}}$ of the silica fiber. The dimensions of ${{\boldsymbol{p}}}_{T}$ are 2 × 2 × 2× 2. ${\widetilde{{\boldsymbol{\sigma }}}}_{T}$ is a 2 × 2 tensor, whose elements can be explicitly written as

A4 $${\widetilde{\sigma }}_{{Tij},1,2}=-{n}_{0}^{4}\sum _{k,l}{p}_{T,{ijkl}}{I}_{{kl},1,2}$$

Here $k,l$ also scan over $x$ or $y$. The transverse profile of the electro-strictive force per unit volume is given by (see Eq. (6) and Eq. (7)):

A5 $${\vec{f}}_{1,2}=-{\boldsymbol{\nabla }}\cdot {\widetilde{{\boldsymbol{\sigma }}}}_{T1,2}$$

A6 $${f}_{i,1,2}=-\sum _{j}\frac{\partial }{\partial j}{\widetilde{\sigma }}_{{Tij},1,2}$$

The normalized profile of material displacement [m^−1^] in mode ${{TR}}_{{pm}}$ can expressed as

A7 $${\vec{u}}_{{pm}}\left(x,y\right)={u}_{x,{pm}}\left(x,y\right)\hat{{\boldsymbol{x}}}{\boldsymbol{+}}{u}_{y,{pm}}\left(x,y\right)\hat{{\boldsymbol{y}}}$$

The spatial overlap integral of electro-strictive stimulation through optical modes 1 and 2 and acoustic mode ${{TR}}_{{pm}}$ equals:

A8 $${Q}_{1,2,{pm}}^{({ES})}=\iint \sum _{i}{f}_{i,1,2}{u}_{i,{pm}}{ds}$$

where ${ds}$ is an infinitesimal area element in the transverse plane. We may write

A9 $$\sum _{i}{f}_{i,1,2}{u}_{i,{pm}}=-\sum _{i}\sum _{j}\frac{\partial }{\partial j}{\widetilde{\sigma }}_{{Tij},1,2}{u}_{i,{pm}}={n}_{0}^{4}\sum _{i}\sum _{j}\frac{\partial }{\partial j}\left(\sum _{k,l}{p}_{T,{ijkl}}{I}_{{kl},1,2}\right){u}_{i,{pm}}={n}_{0}^{4}\sum _{i,j,k,l}{p}_{T,{ijkl}}{u}_{i,{pm}}\frac{\partial }{\partial j}{I}_{{kl},1,2}$$

Leading to

A10 $${Q}_{{pm}}^{({ES})}={n}_{0}^{4}\iint \left(\sum _{i,j,k,l}{p}_{T,{ijkl}}{u}_{i,{pm}}\frac{\partial }{\partial j}{I}_{{kl},1,2}\right){ds}$$

In Eq. (A9) we assume that the tensor ${{\boldsymbol{p}}}_{T}$ is constant across the silica fiber, and neglect small scale differences between core and cladding. Expressing the summation over $j=x,y$ explicitly, and moving the summation outside the integral, we obtain

A11 $${Q}_{1,2,{pm}}^{({ES})}={n}_{0}^{4}\sum _{i,k,l}\iint \left({p}_{T,{ixkl}}{u}_{i,{pm}}\frac{\partial }{\partial x}{I}_{{kl},1,2}+{p}_{T,{iykl}}{u}_{i,{pm}}\frac{\partial }{\partial y}{I}_{{kl},1,2}\right){ds}$$

Next, we address the spatial overlap integral of photoelastic scattering. The normalized strain associated with the material displacement is a 2 × 2 tensor:

A12 $${{\boldsymbol{s}}}_{{pm}}={\boldsymbol{\nabla }}{\vec{u}}_{{pm}}$$

With elements:

A13 $${s}_{{ij},{pm}}=\frac{\partial }{\partial j}{u}_{i,{pm}}$$

Strain leads to photoelastic perturbations in the dielectric tensor of the fiber. The transverse dependence of the perturbations is in the form of a tensor ${{\boldsymbol{\mu }}}_{T,{pm}}$ [41]:

A14 $${{\boldsymbol{\mu }}}_{T,{pm}}=-{n}_{0}^{4}{{\boldsymbol{p}}}_{T}:{{\boldsymbol{s}}}_{{pm}}$$

A15 $${\mu }_{T,{ij},{pm}}=-{n}_{0}^{4}\sum _{k,l}{p}_{T,{ijkl}}{s}_{{kl},{pm}}$$

The spatial overlap integral of photoelastic scattering between optical modes 1 and 2 through acoustic mode ${{TR}}_{{pm}}$ is given by (see Eq. (21)):

A16 $${Q}_{1,2,{pm}}^{({PE})}=\iint \sum _{i,j}{\mu }_{T,{ij},{pm}}{I}_{{ij},1,2}{ds}$$

This integrand can be rearranged in the following form

A17 $$\sum _{i,j}{\mu }_{T,{ij},{pm}}{I}_{{ij},1,2}=-{n}_{0}^{4}\sum _{i,j}\sum _{k,l}{p}_{T,{ijkl}}{s}_{{kl},{pm}}{I}_{{ij},1,2}=-{n}_{0}^{4}\sum _{i,j,k,l}{p}_{T,{ijkl}}{I}_{{ij},1,2}\frac{\partial }{\partial l}{u}_{k,{pm}}$$

With a change of indices ${i}^{{\prime} }=k,{j}^{{\prime} }=l,{k}^{{\prime} }=i,{l}^{{\prime} }=j$, we obtain

A18 $$\sum _{i,j}{\mu }_{T,{ij},{pm}}{I}_{{ij},1,2}=-{n}_{0}^{4}\sum _{k{\prime} ,l{\prime} ,i{\prime} ,j{\prime} }{p}_{T,k{\prime} l{\prime} i{\prime} j{\prime} }{I}_{{k}^{{\prime} }{l}^{{\prime} },1,2}\frac{\partial }{\partial j{\prime} }{u}_{i{\prime} ,{pm}}$$

(The prime superscripts are omitted hereunder). The tensor ${{\boldsymbol{p}}}_{T}$ is symmetric, hence ${p}_{T,{klij}}={p}_{T,{ijkl}}$. The photoelastic overlap integral is therefore brought to the following form

A19 $${Q}_{1,2,{pm}}^{({PE})}=-{n}_{0}^{4}\sum _{i,j,k,l}\iint {p}_{T,{ijkl}}{I}_{{kl},1,2}\frac{\partial }{\partial j}{u}_{i,{pm}}{ds}$$

Expressing once again the summation with respect to index $j$ in a direct form, we obtain

A20 $${Q}_{1,2,{pm}}^{({PE})}=-{n}_{0}^{4}\sum _{i,k,l}\iint \left({p}_{T,{ixkl}}{I}_{{kl},1,2}\frac{\partial }{\partial x}{u}_{i,{pm}}+{p}_{T,{iykl}}{I}_{{kl},1,2}\frac{\partial }{\partial y}{u}_{i,{pm}}\right){ds}$$

Integration in parts yields:

A21 $${Q}_{1,2,{pm}}^{({PE})}={n}_{0}^{4}\sum _{i,k,l}\iint \left({p}_{T,{ixkl}}{u}_{i,{pm}}\frac{\partial }{\partial x}{I}_{{kl},1,2}+{p}_{T,{iykl}}{u}_{i,{pm}}\frac{\partial }{\partial y}{I}_{{kl},1,2}\right){ds}-{n}_{0}^{4}\sum _{i,k,l}\left({\oint }_{L}{p}_{T,{ixkl}}{u}_{i,{pm}}{I}_{{kl},1,2}{dy}+{\oint }_{L}{p}_{T,{iykl}}{u}_{i,{pm}}{I}_{{kl},1,2}{dx}\right)$$

The first two terms in Eq. (A21) equal ${Q}_{\mathrm{1,2},{pm}}^{({ES})}$ (see Eq. A(11)). The last two terms are integrals carried out over the outer circumference of the cladding cross-section. At the cladding edge, ${I}_{{kl},\mathrm{1,2}}=0$. These two terms therefore vanish, and we obtain

A22 $${Q}_{1,2,{pm}}^{({PE})}={Q}_{1,2,{pm}}^{({ES})}$$
